# Draft Genome Sequence of Sphingorhabdus sp. Strain EL138, a Metabolically Versatile Alphaproteobacterium Isolated from the Gorgonian Coral Eunicella labiata

**DOI:** 10.1128/genomeA.00142-18

**Published:** 2018-03-01

**Authors:** Sandra Godinho Silva, Asunción Lago-Lestón, Rodrigo Costa, Tina Keller-Costa

**Affiliations:** a IBB—Institute for Bioengineering and Biosciences, Department of Bioengineering, Instituto Superior Técnico Universidade de Lisboa, Lisbon, Portugal; b Department of Bioengineering, Instituto Superior Técnico Universidade de Lisboa, Lisbon, Portugal; c Centro de Investigación Científica y de Educación Superior de Ensenada (CICESE), Ensenada, Mexico

## Abstract

Here, we report the draft genome sequence of Sphingorhabdus sp. strain EL138, an alphaproteobacterium that shows potential to degrade polycyclic aromatic compounds and to cope with various heavy metals and antibiotics. Moreover, the strain, isolated from the gorgonian coral Eunicella labiata, possesses several genes involved in the biosynthesis of polyphosphates, polyketides, and terpenoids.

## GENOME ANNOUNCEMENT

The genus Sphingorhabdus, recently described in 2013 by Jogler et al. (1), belongs to the widespread alphaproteobacterium family Sphingomonadaceae, members of which are known for their ability to degrade several polycyclic aromatic compounds and xenobiotics (2, 3). There are currently eight validly named species (4) of the genus Sphingorhabdus, but only five with available genome sequences. These bacteria have been found in coastal sediments and freshwater lakes (4) and were also recently described in association with a soft coral (5). However, we currently lack in-depth knowledge of their patterns of association with eukaryotic hosts. Here, we report the first genome sequence of a Sphingorhabdus strain retrieved from a gorgonian coral.

Strain EL138 was isolated from the soft coral Eunicella labiata (Octocorallia, Gorgoniidae), which was sampled at a depth of 18 m off the coast of Faro, South Portugal (36°58′47.2″N, 7°59′20.8″W) (5). After sampling, the E. labiata sample was immediately transported to the laboratory, and cell suspensions, prepared as described previously (5, 6), were inoculated onto 1:2 (vol/vol) marine broth medium containing 1.5% agar medium for 4 weeks at 18°C. Genomic DNA was extracted using the Wizard genomic DNA purification kit (Promega, Madison, USA) from a pure culture freshly grown in marine broth (1:2 vol/vol) and was further sequenced on a MiSeq platform (Illumina), as described elsewhere (7, 8). The sequence output was 803 Mb, consisting of 2 × 1,334,335 paired-end reads of 301 bp and corresponding to 219× coverage of the genome. Sequence reads were assembled *de novo* into 4 Sphingorhabdus contigs using the NGen DNA assembly software (DNAStar, Inc.). Annotation was conducted with the Rapid Annotations using Subsystems Technology (RAST) server version 2.0 (9). The antiSMASH platform was used to identify biosynthetic gene clusters.

The genome is composed of 3,662,861 bp, with a calculated GC content of 53.1%. It possesses 3,622 coding sequences and 44 RNAs. The closest type strain, with a 99.22% 16S rRNA gene sequence similarity, is Sphingorhabdus litoris (previously Sphingopyxis litoris) strain FR1093, which was previously isolated from surface seawater (1, 10). Genome annotation showed the presence of genes encoding polyphosphate kinase (EC 2.7.4.1) and exopolyphosphatase (EC 3.6.1.11) involved in the synthesis and modification of highly energetic polyphosphates. This suggests that strain EL138 has a role in phosphorus provision for its gorgonian host, a phenomenon recently described for sponge bacterial symbionts (11). In addition, multiple genes associated with homeostasis and resistance to heavy metals, such as copper, cobalt, zinc, cadmium, and mercury, as well as resistance to antibiotics (including 11 beta-lactamase and 15 multidrug resistance efflux pump genes) were found. Versatile biodegradation capabilities were revealed through the presence of 24 genes involved in the breakdown of *N*-heterocyclic aromatic compounds (via EC 1.3.99.16) and other (potentially polycyclic) aromatic hydrocarbons (e.g., via EC 1.14.13.1 and EC 3.1.1.24). Furthermore, strain EL138 harbors a terpenoid gene cluster that shares 75% similarity with that encoding the carotenoid food dye astaxanthin, which is known for its anti-inflammatory and antitumor activities (12). In addition, a putative type III polyketide synthase gene cluster (41-kb) of the chalcone synthase type was identified.

### Accession number(s).

The genome sequence of Sphingorhabdus sp. EL138 has been deposited in the European Nucleotide Archive (ENA) under the sequence accession numbers OGVD01000001 to OGVD01000004. The study identification number is PRJEB24502, and the sample identification number is ERS2111500.
